# Prospectively Accelerated T2-Weighted Imaging of the Prostate by Combining Compressed SENSE and Deep Learning in Patients with Histologically Proven Prostate Cancer

**DOI:** 10.3390/cancers14235741

**Published:** 2022-11-22

**Authors:** Felix N. Harder, Kilian Weiss, Thomas Amiel, Johannes M. Peeters, Robert Tauber, Sebastian Ziegelmayer, Egon Burian, Marcus R. Makowski, Andreas P. Sauter, Jürgen E. Gschwend, Dimitrios C. Karampinos, Rickmer F. Braren

**Affiliations:** 1 Institute of Diagnostic and Interventional Radiology, School of Medicine, Technical University of Munich, Ismaninger Str. 22, 81675 Munich, Germany; 2 Philips GmbH, Röntgenstrasse 22, 22335 Hamburg, Germany; 3 Department of Urology, Technical University of Munich, Ismaninger Str. 22, 81675 Munich, Germany; 4 Philips Healthcare, Veenpluis 4-6, Building QR-0.113, 5684 Best, The Netherlands

**Keywords:** prostate cancer, MRI, deep learning, compressed sensing

## Abstract

**Simple Summary:**

Since prostate MRI is increasingly applied and yet limited by long acquisition times, we prospectively investigated the performance of a novel reconstruction algorithm combining compressed sensing, parallel imaging and deep learning (C-SENSE AI) in patients with histologically proven prostate cancer. Highly accelerated T2w images were compared to clinical standard-of-reference T2w images. C-SENSE AI enabled a 58% acceleration in T2w imaging of the prostate while obtaining significantly better image quality and tumor detection. C-SENSE AI seems particularly interesting in view of the need for accelerated prostate MRI (e.g., in screening protocols) with preserved high image quality.

**Abstract:**

Background: To assess the performance of prospectively accelerated and deep learning (DL) reconstructed T2-weighted (T2w) imaging in volunteers and patients with histologically proven prostate cancer (PCa). Methods: Prospectively undersampled T2w datasets were acquired with acceleration factors of 1.7 (reference), 3.4 and 4.8 in 10 healthy volunteers and 23 patients with histologically proven PCa. Image reconstructions using compressed SENSE (C-SENSE) and a combination of C-SENSE and DL-based artificial intelligence (C-SENSE AI) were analyzed. Qualitative image comparison was performed using a 6-point Likert scale (overall image quality, noise, motion artifacts, lesion detection, diagnostic certainty); the T2 and PI-RADS scores were compared between the two reconstructions. Additionally, quantitative image parameters were assessed (apparent SNR, apparent CNR, lesion size, line profiles). Results: All C-SENSE AI-reconstructed images received a significantly higher qualitative rating compared to the C-SENSE standard images. Analysis of the quantitative parameters supported this finding, with significantly higher aSNR and aCNR. The line profiles demonstrated a significantly steeper signal change at the border of the prostatic lesion and the adjacent normal tissue in the C-SENSE AI-reconstructed images, whereas the T2 and PI-RADS scores as well as the lesion size did not differ. Conclusion: In this prospective study, we demonstrated the clinical feasibility of a novel C-SENSE AI reconstruction enabling a 58% acceleration in T2w imaging of the prostate while obtaining significantly better image quality.

## 1. Introduction

Prostate cancer (PCa) is the second most common malignancy in men worldwide, with over 1.2 million new cases per year [1]. Multiparametric magnetic resonance imaging (mpMRI) outperforms other imaging modalities in the visualization of the prostate and is increasingly applied owing to its high significance for lesion detection, preoperative staging and biopsy guidance [2,3]. T2-weigthed (T2w) and diffusion-weighted imaging (DWI) have a central role in lesion detection and characterization [4]. Yet, particularly, the T2w sequences contribute to the very long examination time required for prostate MRI.

In the past, parallel imaging (PI) and more recently sparse sampling methods, such as compressed sensing (CS), have emerged as powerful techniques to accelerate MRI acquisition across a variety of organs, including the prostate [5,6,7,8,9,10,11,12]. Compressed SENSE (C-SENSE) combines both acceleration techniques, compressed sensing (CS) and PI, using SENSE (SENSitivity Encoding) and results in better image quality compared to PI alone [13].

Recent studies proposed the application of machine learning approaches in MRI image reconstruction to ameliorate image quality and further accelerate image acquisition [14,15,16,17]. Promising results were also reported for the MRI of the prostate. However, they warrant further evaluation, as previous studies relied on retrospectively undersampled data from fully sampled acquisitions [18,19].

Considering the unprecedented demand for prostate MRI, the long acquisition times and the increasing availability of deep learning (DL) approaches in MRI, we herein evaluated the prospective image acceleration and application of a reconstruction algorithm combining a DL algorithm, named Adaptive-CS-Net, with the C-SENSE framework, to enable highly accelerated T2w imaging in patients with histologically proven PCa to specifically investigate the performance of this new technique in the presence of pathologies.

## 2. Materials and Methods

### 2.1. Study Design and Patient Cohort

This was a prospective single-center feasibility study. We aimed to prove the feasibility of the applied deep learning (DL) reconstruction algorithm in 10 healthy volunteers before including patients with PCa. The algorithm used in this study has previously only been applied in musculoskeletal imaging, and the optimal compressed sense factor can vary when imaging different anatomic regions. Hence, we decided to test the applicability of this algorithm as well as of the investigated CS factors in healthy volunteers first. Patients were referred for biopsy-proven PCa to the urology unit of our tertiary hospital. All patients who were scheduled to undergo prostatectomy and with a clinical indication for a pre-operative MRI were offered participation in this study. The exclusion criteria were contraindications to undergo an MRI examination and previous prostate operations. The volunteers had no previous history of a known prostate disease. The study was conducted in accordance with the Declaration of Helsinki. Approval by the local ethics committee (protocol Nr. 106/20 S-SR, 102/21 S-EB) was given, and written informed consent was obtained from every patient.

### 2.2. Clinical Data

The following clinical data were obtained for all patients using the hospital’s information system: age at diagnosis, prostate-specific antigen (PSA) level (ng/mL), tumor grading and tumor staging.

### 2.3. Data Acquisition

All MRI datasets were acquired on a whole-body 3T MRI system (Philips Ingenia Elition X, Philips Healthcare, Best, Netherlands) using a combination of a 16-channel torso coil array and an inbuilt table posterior 12-channel coil array. No endorectal coils were applied.

In all participants, an axial T2w turbo-spin-echo (TSE) sequence as well as an axial 2D DWI sequence were performed. ADC maps were generated from b values of 50, 500 and 1000 s/mm^2^. The T2w scans were accelerated by prospective, pseudorandom and density-weighted k-space sampling with higher sampling density towards the k-space center. The following acceleration factors were applied: 1.7, 3.4 and 4.8. Further imaging parameters are provided in Table 1. Hyoscine butylbromide (HBB) was applied in all patients with no contraindications. The volunteers did not receive HBB.

### 2.4. Data Reconstruction

To improve the performance of image reconstruction using DL, the C-SENSE technique was combined with an Adaptive-CS-Network as presented by Pezzotti et al. (C-SENSE AI) [20]. In short, the Adaptive-CS-Network mimics the Iterative Shrinkage-Thresholding Algorithm (ISTA) approach presented by Zhang and Ghanem and integrates multiscale sparsification in a problem-specific learnable manner [21]. This sparsifying approach based on a convolutional neural network (CNN) was combined with the image reconstruction approach of C-SENSE, which ensures data consistency and incorporates prior knowledge such as coil sensitivity distribution and location of the image background. In this approach, the Adaptive-CS-Network replaces the wavelet transform as the sparsifying transform in the C-SENSE algorithm, combining parallel imaging, compressed sensing and deep learning in a single, iterative image reconstruction algorithm. Other than presented by Pezzotti et al., the Adaptive-CS-Network employed in this work was trained with about 740.000 MR images from both 1.5T and 3T and various anatomies and contrasts [20]. The algorithm was optimized to allow execution on standard reconstruction hardware and was provided as a work-in-progress package. A dedicated graphics processing unit (GPU) (NVIDIA QUADRO RTX 5000) was integrated in the reconstruction hardware. All images were reconstructed online directly at the scanner. Accelerated T2w TSE data were additionally reconstructed with the C-SENSE AI technique. The reconstruction times were below 1 min.

### 2.5. T2w Imaging

As routinely performed in our institution, the T2w imaging sequence with an acceleration factor of 1.7 was reconstructed with C-SENSE (T2w_C-SENSE1.7_) and served as a standard of reference. Additionally, the T2w imaging sequences with acceleration factors of 1.7, 3.4 and 4.8 were reconstructed using C-SENSE AI (T2w_C-SENSE AI1.7_, T2w_C-SENSE AI3.4_ and T2w_C-SENSE AI4.8_, respectively).

### 2.6. Image Analysis

To analyze the performance of the C-SENSE AI reconstructions for T2w imaging the standard of reference T2w_C-SENSE1.7_ was compared to T2w_C-SENSE AI1.7_, T2w_C-SENSE AI3.4_ and T2w_C-SENSE AI4.8_.

### 2.7. Qualitative Image Analysis

Qualitative image analysis was performed independently and separately by four radiologists with 3 to 7 years of experience. All images from both patients and volunteers were analyzed using OsiriX (OsiriX DICOM viewer, 11.0 OsiriX Foundation). Qualitative assessment was performed using a 6-point Likert-scale, with 1 indicating the worst, and 6 the best score with regard to overall image quality, noise, motion artifacts, image sharpness, lesion detection and diagnostic certainty. Detailed parameters are provided in the Appendix A. Furthermore, the T2 and PI-RADS scores of the highest scored lesion based on T2w and DWI were compared between the standard of reference T2w_C-SENSE1.7_ and T2w_C-SENSE AI1.7_, T2w_C-SENSE AI3.4_ and T2w_C-SENSE AI4.8_. To minimize the risk of inherent bias, the T2 and PI-RADS scores were assessed in a second reading session after 2 weeks, with images being presented in a random order. The T2 and PI-RADS scores and qualitative lesion assessment were performed only in patients.

### 2.8. Quantitative Image Analysis

Quantitative image analysis was performed by one radiologist (3 years of experience) under the supervision of a radiologist with 7 years of experience. The apparent signal-to-noise ratio (aSNR) for the T2w sequences was calculated in all healthy volunteers. Using the standard of reference T2w_C-SENSE1.7_, a 5 mm circular region of interest (ROI) was placed in the left and right anterior peripheral zone (PZa) and transferred to all other sequences (T2w_C-SENSE AI1.7_, T2w_C-SENSE AI3.4,_ T2w_C-SENSE AI4.8_). Mean values were calculated for the prostate and the internal obturator muscles.

The aSNR was calculated as follows:

$$\mathrm{aSNR}\;=\frac{{\mathrm{SI}}_{PZa}}{{\mathrm{SD}}_{muscle}}$$

To determine the apparent contrast-to-noise ratio (aCNR), a 5 mm circular ROI was placed in the largest prostatic lesion and the adjacent normal prostate parenchyma in the T2w_C-SENSE1.7_ and again transferred as described above. The aCNR was calculated as follows:

$$\mathrm{aCNR}\;=\frac{\left({\mathrm{SI}}_{lesion}-{\mathrm{SI}}_{normal}\right)}{{\mathrm{SD}}_{normal}}$$

In addition, to investigate image sharpness, the FIJI’s “Line profile” tool was used [22]. A line was manually drawn in the largest lesion at its widest diameter in the T2w images. Signal intensities along the line profile were expressed as percentages of the lowest intensity inside the lesion. Based on the change per mm at the lesion boundary, a slope profile was calculated. Furthermore, an ROI was placed over the largest lesion in in each sequence to determine the lesion size at its largest axial extent in each sequence.

### 2.9. Statistical Analysis

The Shapiro–Wilk test was applied to test for a normal distribution of the data. A paired *t*-test was used for a mean comparison of normally distributed variables, while the Wilcoxon test was used for variables without normal distribution.

Inter-rater agreement was calculated using Fleiss’ kappa and considered as slight: κ = 0.00 − 0.20; fair: κ = 0.21 − 0.40, moderate: κ = 0.41 − 0.60, substantial for κ = 0.61 − 0.80, and almost perfect: κ = 0.81 − 1.00 [23]. *p*-values ≤ 0.05 were considered statistically significant. All statistics were performed in IBM SPSS (IBM Corp.), version 25.

## 3. Results

Between January and April 2021, a total of 10 healthy volunteers (28.1 ± 3.8 years) and 23 patients (64.4 ± 6.2 years) were included. Seven patients did not receive HBB due to contraindications. The patient inclusion flowchart can be found in the Appendix A. Detailed patient characteristics are displayed in Table 2.

### 3.1. Determination of Suitable Acceleration Factors

Two acceleration factors were chosen, one rather conservative acceleration factor of 3.4 and a higher acceleration factor of 4.8, with the aim to explore the limits of the reconstruction technique, without a too extensive prolongation of the patient scans. Investigation of further acceleration factors was not considered reasonable with regard to the available examination time.

### 3.2. Qualitative Analysis

#### 3.2.1. Image Quality

**Figure 1:**
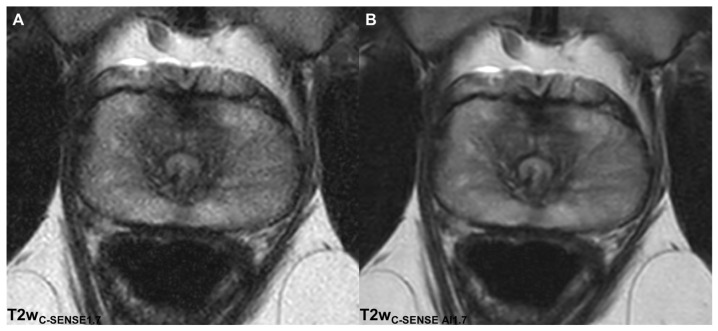
Axial T2w images in a 30-year-old volunteer acquired with an acceleration factor of 1.7. A significantly higher noise level is noted in the C-SENSE (**A**) compared to the C-SENSE AI (**B**) reconstruction.

**Figure 2:**
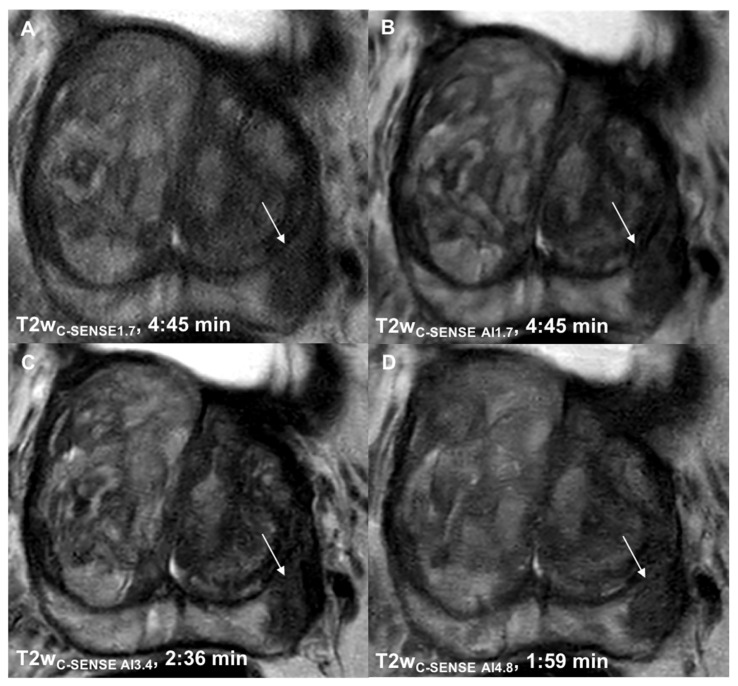
Axial T2w images of the standard-of-reference sequence (**A**) and the Compressed Sense accelerated study sequences in a 60-year-old patient with a PI-RADS 5 lesion (arrow) in the left PZpl. Due to contraindications, no HBB was administered in this patient. Significant noise reduction can be seen in T2w_C-SENSE AI1.7_ (**B**) compared to T2w_C-SENSE1.7_. Superior image quality was obtained even after two-fold (**C**) and three-fold (**D**) acceleration of the acquisition time in the DL-enhanced study sequences.

Image quality was rated significantly higher in T2w_C-SENSE AI1.7_ (5.06 ± 0.79, *p* < 0.00001), T2w_C-SENSE AI3.4_ (5.34 ± 0.69, *p* < 0.00001) and T2w_C-SENSE AI4.8_ (4.28 ± 0.51, *p* < 0.00001) compared to T2w_C-SENSE1.7_ (3.05 ± 0.76) (Table 3, Figure 1 and Figure 2). Fleiss’ kappa revealed a high inter-rater agreement (κ = 0.81 − 0.83).

#### 3.2.2. Noise

Noise was found to be significantly reduced in the AI-reconstructed study sequences T2w_C-SENSE AI1.7_ (4.63 ± 0.86, *p* < 0.00001), T2w_C-SENSE AI3.4_ (4.69 ± 0.81, *p* < 0.00001) and T2w_C-SENSE AI4.8_ (4.09 ± 0.68, *p* < 0.00001) compared to the standard-of-reference T2w_C-SENSE1.7_ (3.19 ± 0.68) (Table 3, Figure 1 and Figure 2). Fleiss’ kappa revealed a high inter-rater agreement (κ = 0.82 − 0.85).

#### 3.2.3. Motion Artifacts

Motion artifacts did not differ between the standard-of-reference sequence (T2w_C-SENSE AI1.7_ 3.53 ± 0.87, *p* = 0.39) and the T2w_C-SENSE AI1.7_ sequence (3.59 ± 0.91). However, a significant reduction in motion artifacts was found for both accelerated sequences, T2w_C-SENSE AI3.4_ (4.91 ± 0.67, *p* < 0.00001) and T2w_C-SENSE AI4.8_ (5.06 ± 0.68, *p* < 0.00001) (Table 3). Fleiss’ kappa revealed a substantial to high inter-rater agreement (κ = 0.78 − 0.84).

#### 3.2.4. Image Sharpness

Image sharpness was rated significantly higher in the AI-reconstructed T2w_C-SENSE AI1.7_ (4.74 ± 0.61, *p* < 0.00001), T2w_C-SENSE AI3_ (4.72 ± 0.62, *p* < 0.00001) and T2w_C-SENSE AI4.8_ (4.66 ± 0.53, *p* < 0.00001) compared to T2w_C-SENSE1.7_ (3.13 ± 0.72), with high inter-rater agreement (κ = 0.86 − 0.91) (Table 3, Figure 1 and Figure 2).

#### 3.2.5. Lesion Detection and Diagnostic Certainty

T2w_C-SENSE AI1.7_ (4.37 ± 0.99, *p* = 0.000065), T2w_C-SENSE AI3.4_ (5.09 ± 0.78, *p* < 0.00001) and T2w_C-SENSE AI4.8_ (4.91 ± 0.72, *p* < 0.00001) led to a significant improvement in lesion detection compared to T2w_C-SENSE1.7_ (3.7 ± 0.62). Furthermore, diagnostic certainty was significantly higher in T2w_C-SENSE AI1.7_ (4.61 ± 1.17, *p* = 0.0014), T2w_C-SENSE AI3.4_ (4.96 ± 0.85, *p* < 0.00001) and T2w_C-SENSE AI4.8_ (4.74 ± 0.89, *p* = 0.000096) compared to T2w_C-SENSE1.7_ (3.57 ± 1) (Table 3). Fleiss’ kappa revealed a substantial to high inter-rater agreement regarding both lesion detection (κ = 0.79 − 0.83) and diagnostic certainty (κ = 0.78 − 0.81)

#### 3.2.6. T2 and PI-RADS Scores

A T2 score of 4 was the most frequent category (*n* = 13, 56%), followed by a score of 3 (*n* = 6, 26%) and a score of 5 (*n* = 4, 17%). The T2 scores did not differ significantly between the standard-of-reference dataset and the study sequences T2w_C-SENSE AI1.7_ (*p* = 0.81), T2w_C-SENSE AI3.4_ (*p* = 0.82) and T2w_C-SENSE AI4.8_ (*p* = 0.81). Fleiss’ kappa revealed an almost perfect inter-rater agreement (κ = 0.81 − 0.89). A PI-RADS score of 4 was the most frequent category (*n* = 14, 61%), followed by a score of 3 (*n* = 6, 26%) and a score of 5 (*n* = 3, 13%). The PI-RADS scores did not differ significantly between the standard of reference dataset and the study sequences T2w_C-SENSE AI1.7_ (*p* = 0.81), T2w_C-SENSE AI3.4_ (*p* = 0.82) and T2w_C-SENSE AI4.8_ (*p* = 0.81).

### 3.3. Quantitative Analysis

#### 3.3.1. aSNR

In 10 healthy volunteers, the aSNR for T2w was evaluated for the right and left prostate lobe, and the mean was calculated. The aSNR was found to be significantly higher in T2w_C-SENSE AI1.7_ (7.68 ± 1.5, *p* = 0.0004), T2w_C-SENSE AI3.4_ (6.61 ± 1.78, *p* = 0.0029) and T2w_C-SENSE AI4.8_ (5.79 ± 0.72, *p* = 0.00027) compared to T2w_C-SENSE1.7_ (4.3 ± 0.47).

#### 3.3.2. aCNR

In all 23 patients, the aCNR measurement was performed in the largest detectable lesion. The aCNR was significantly higher in T2w_C-SENSE AI1.7_ (7.74 ± 2.96, *p* = 0.00029), T2w_C-SENSE AI3.4_ (9.01 ± 4.2, *p* = 0.00016) and T2w_C-SENSE AI4.8_ (6.72 ± 2.84, *p* = 0.006) compared to T2w_C-SENSE1.7_ (4.57 ± 1.75).

#### 3.3.3. Image Sharpness

**Figure 3:**
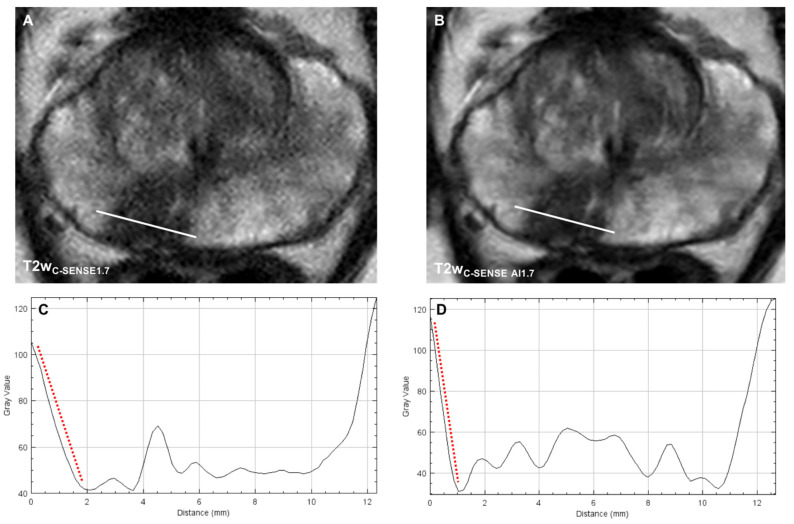
Axial T2w images of a PI-RADS 4 lesion acquired with the standard-of-reference (**A**) and the DL-enhanced study sequences (**B**). The line profile plots of the indicated location demonstrate a significantly steeper signal change at the border of the lesion in the T2w_C-SENSE AI1.7_ (**C**) compared to the T2w_C-SENSE1.7_ (**D**) indicating a sharper lesion demarcation.

**Figure 4:**
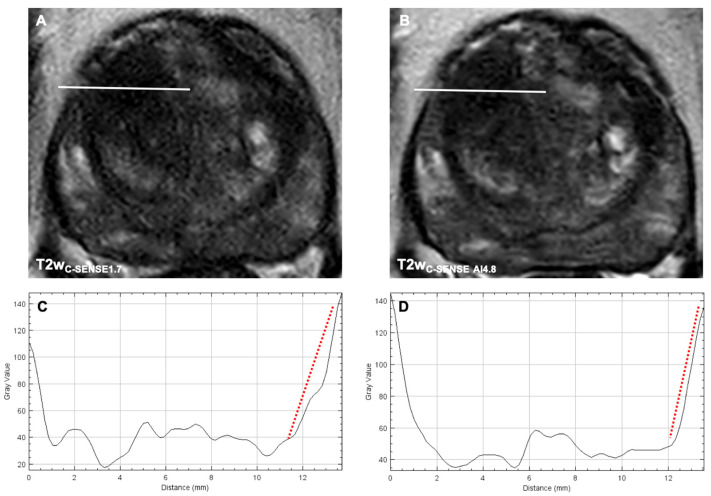
Axial T2w images of a PI-RADS 4 lesion acquired with the standard-of-reference sequence at a CS-factor of 1.7 (**A**) and the DL-enhanced study sequence at a CS-factor of 4.8 (**B**). Line profile plots of the indicated location, demonstrating a steeper signal change at the border of the lesion in the T2w_C-SENSE AI14.8_ (**C**) compared to the T2w_C-SENSE1.7_ (**D**). This indicates a sharper lesion demarcation despite the significantly reduced acquisition time.

The analysis of the line profile slope through the prostatic lesion revealed a significantly steeper signal drop at the border of the prostatic lesion in T2w_C-SENSE AI1.7_ (−40.3 ± 1.23%, *p* = 0.000049), T2w_C-SENSE AI3.4_ (−52.4 ± 11.7%, *p* < 0.00001) and T2w_C-SENSE AI4.8_ (−38.4 ± 9.7%, *p* = 0.00003) compared to T2w_C-SENSE1.7_ (−24.1 ± 8.3%) (Figure 3 and Figure 4).

#### 3.3.4. Lesion Size

Lesion size did not differ among the study sequences when compared to the standard-of-reference dataset for both T2w images (T2w_C-SENSE1.7_ 167.53 mm^2^; T2w_C-SENSE AI1.7_ 165.25 mm^2^, *p* = 0.48; T2w_C-SENSE AI3.4_ 161.88 mm^2^, *p* = 0.49 and T2w_C-SENSE AI4.8_: 161.88 mm^2^, *p* = 0.49).

## 4. Discussion

In this prospective feasibility study, we assessed a novel reconstruction algorithm, combining CS, PI and DL (C-SENSE AI), for highly accelerated T2w imaging of the prostate. C-SENSE AI reconstructions provided superior image quality, better lesion conspicuity and, simultaneously, a significant reduction of 58% of the scan time compared to C-SENSE- and SENSE-based image reconstruction.

Prostate mpMRI has emerged as a cornerstone in patient management and risk stratification, reflected by an unprecedented number of examinations performed [24]. The T2w sequences represent a mainstay for lesion characterization in the PI-RADS protocol, yet constitute a major part of the long examination time. CS has emerged as a powerful technique to reduce the acquisition time especially when combined with PI [12,25,26]. However, the advantages of higher acceleration factors have been counterbalanced particularly by the simultaneous degradation of image quality.

Recently, artificial intelligence algorithms have been introduced as a promising tool to further reduce the examination times and ameliorate image quality, e.g., in CS-accelerated musculoskeletal, vascular and abdominal MRI [16,17,27]. Initially, in prostate MRI, machine learning approaches have been applied mostly for lesion detection and classification [28,29,30]. However, recent studies also focused on scan acceleration in prostate MRI [18,31,32,33].

In our study, we focused on the application of C-SENSE AI in patients with histologically proven prostate cancer.

C-SENSE AI reconstructions enabled an increase of the CS factor to 3.4 and 4.8, reducing the acquisition time by 45% and 58%, respectively. Furthermore, better lesion detection, higher diagnostic certainty and higher CNR and SNR were found in both T2w_C-SENSE AI3.4_ and T2w_C-SENSE AI4.8_, compared to the standard of reference. However, perceived mean image quality and noise levels were higher in T2w_C-SENSE AI1.7_ and T2w_C-SENSE AI3.4_ compared to T2w_C-SENSE AI4.8_.

This might indicate limited denoising capability at even higher acceleration factors and warrants further investigation. In summary, these findings suggest T2w_C-SENSE AI4.8_ as the most suitable sequence for lesion detection and characterization.

Likewise, perceived motion artifacts were significantly reduced in T2w_C-SENSE AI3.4_ and T2w_C-SENSE AI4.8_ compared to T2w_C-SENSE1.7_ as well as T2w_C-SENSE AI1.7_. This finding was supported by the quantitative analysis of the slope line profiles. We attributed this to the faster image acquisition and thus reduced motion susceptibility. It is of note that the increase in image quality and lesion detection and the reduction in motion artifacts in T2w_C-SENSE AI3.4_ and T2w_C-SENSE AI4.8_ were particularly remarkable in a subset of eight patients who did not receive HBB. In many institutions, HBB is applied to minimize motion artifacts due to bowel movement [34]. However, other studies question the meaningfulness of HBB application in prostate MRI [35]. Our results indicate that highly accelerated MRI in combination with C-SENSE AI reconstruction could be of particular interest in patients with contraindications for the administration of HBB.

Our study is the first to assess the combination of DL, CS and PI in a single reconstruction algorithm in MRI of the prostate. Two previous studies from one group evaluated the feasibility of DL and PI in T2w prostate MRI, reporting good image quality and a similar acquisition time compared to our work [31,32]. However, in PI, the maximum achievable acceleration is restricted particularly by the coil geometry and is in most applications limited to the factor of 2 to 4 [15,36,37]. A loss of SNR proportional to the acceleration factor is a major penalty of PI [36]. In contrast, CS is less prone to SNR deterioration at high acceleration factors due to its inherent iterative denoising capability [17,25]. Combining CS and PI has been reported to accelerate the acquisition substantially and ameliorate image quality compared to PI alone [13,26,38]. One of the strengths of our work is the prospective design. Previous studies demonstrated the potential value of DL-based approaches for retrospectively accelerated MRI of the prostate MRI and the knee. [17,18] However, to study the clinical performance of any acceleration method, it is essential to investigate the effects of prospective undersampling on image quality. Interactions between the actual k-space sampling scheme and physiological motion or eddy currents related to pseudo-random sampling might impact the quality of the acquired k-space data. Therefore, it is required to investigate the clinical applicability and robustness as well as the reliable detection of pathologies in prospectively highly accelerated MR imaging. Gassenmeier et al., recently investigated DL-based prospectively undersampled data acquisition in prostate MRI [31,32]. However, in their studies, accelerated image acquisition was achieved by reducing the number of signal averages (NSA). Although this is an effective method, it is only applicable for sequences which use multiple NSA. Additionally, multiple NSA are often used to mitigate the effects of physiological or patient motion, and therefore, a reduction of NSA might increase the motion sensitivity of a sequence. To this end, the investigation of prospectively undersampled scans is important.

According to the current PI-RADS v2.1 protocol, an axial T2w acquisition is mandatory, accompanied, at a minimum, by one additional orthogonal plane [4]. In clinical routine, most institutions perform T2w imaging in three planes.

However, previous studies demonstrated the non-inferiority of short MR protocols consisting of an axial T2w and DWI, compared to the conventional mpMRI protocol [39,40]. Weiss et al., demonstrated a reduced MRI protocol comprising an axial T2w and DWI (b0/1500 s/mm^2^) screening protocol to be comparable to a fully diagnostic mpMRI [40]. Combining the AI-reconstructed axial T2w_C-SENSE AI4.8_ (acq. time 1:59) and DWI_b1500_ (total acq. time 3:48 min) as performed in our study resulted in an acquisition time around six minutes, which would be suitable as a rapid screening protocol. Potentially enabling higher patient throughput, highly accelerated MRI sequences as herein proposed could help to transfer rapid screening protocols into the clinical practice.

### Limitations

Our single-center study included only a small patient cohort with PCa. Other prostatic pathologies were not evaluated. Further, prospective multicenter studies enabling the assessment of larger patient cohorts and various prostatic pathologies should be conducted in the future. Additionally, only axial T2w images were acquired. Further studies should evaluate the value of the herein investigated C-SENSE AI reconstruction technique in a full mpMRI. In addition, we only investigated acceleration factors up to 4.8. Further, prospective studies should elucidate the applicability of even faster image acquisition.

## 5. Conclusions

In conclusion, we here demonstrated the clinical feasibility of a C-SENSE AI reconstruction technique combining DL, CS and PI and enabling scan time reductions of 58% for T2w imaging of the prostate with superior image quality compared to the standard of reference.

## Figures and Tables

**Table 1:** Imaging parameters. T2w: T2-weighted; DWI: diffusion-weighted imaging; Ref: reference; Acc: accelerated; TE: echo time; TR: repetition time; FOV: field of view; Hz: Hertz; SENSE: SENSitivity Encoding; Min: minutes.

Acquisition Parameters		
TE/TR ms	120/4600	82/3000 (b1500: 86/3000)
FOV mm^3^	150 × 150 × 90	160 × 160 × 90
Voxel size mm^3^	0.46 × 0.5 × 3	2 × 2 × 3
Slices	30	30
Bandwith (Hz)	232	1854.2
Acceleration factor	1.7/3.4/4.8	-
Parallel imaging factor (SENSE)	-	3
B values (averages) s/mm^2^	-	50 (3), 500 (2), 1000 (12), 1500 (12)
Scan time (min)	4:45/2:36/1:59	5:12 (b50-1000) 3:48 (b1500)

**Table 2:** Patient characteristics. SD: standard deviation; PZ: peripheral zone; TZ: transition zone; pl: posterolateral; pm: posteromedial, a: anterior; PI-RADS: Prostate imaging-reporting and data system; PSA: prostate-specific antigen.

Parameter	Variable	Value
Age (years)	Mean ± SD	64.4 ± 6.2
Number of lesions	1	12 (52%)
	2	8 (39%)
	3	2 (9%)
Lesion location	PZpl	8 (35%)
	PZpm	4 (17%)
	PZa	6 (26%)
	TZa	5 (22%)
PIRADS score	3	3 (13%)
	4	14 (61%)
	5	6 (26%)
Gleason score	6	1 (4%)
	7a	11 (48%)
	7b	9 (39%)
	9	2 (9%)
Tumor size	pT2a	1 (4%)
	pT2c	13 (57%)
	pT3a	4 (17%)
	pT3b	5 (22%)
Nodal status	pN0	22 (96%)
	pN1	1 (4%)
Metastasis	cM0	23 (100%)
PSA (ng/mL)	Mean ± SD	14.4 ± 18.4

**Table 3:** Summary of the qualitative image quality scores for the T2w sequences. The values are shown as mean ± standard deviation as well as minimum and maximum values in brackets. T2w: T2-weighted; C-SENSE: Compressed SENSE; AI: artificial intelligence.

Category	T2w _C-SENSE1.7_	T2w _C-SENSE AI1.7_	*p*	T2w _C-SENSE AI3.4_	*p*	T2w _C-SENSE AI4.8_	*p*
Image quality	3.05 ± 0.76 (2–5)	5.06 ± 0.79 (3–6)	<0.00001	5.34 ± 0.69 (3–6)	<0.00001	4.28 ± 0.51 (4–6)	<0.00001
Noise	3.19 ± 0.68 (2–5)	4.63 ± 0.86 (3–6)	<0.00001	4.69 ± 0.81 (2–6)	<0.00001	4.09 ± 0.68 (3–6)	<0.00001
Motion Artifacts	3.53 ± 0.87 (2–5)	3.59 ± 0.91 (2–5)	0.39	4.91 ± 0.67 (3–6)	<0.00001	5.06 ± 0.68 (4–6)	<0.00001
Image sharpness	3.13 ± 0.72 (2–5)	4.74 ± 0.61 (3–6)	<0.00001	4.72 ± 0.62 (3–6)	<0.00001	4.66 ± 0.53 (4–6)	<0.00001
Lesion detection	3.7 ± 0.62 (3–5)	4.73 ± 0.99 (3–6)	0.000065	5.09 ± 0.78 (3–6)	<0.00001	4.91 ± 0.72 (4–6)	<0.00001
Diagnostic certainty	3.57 ± 1.01 (2–5)	4.6 ± 1.17 (3–6)	0.0014	4.96 ± 0.85 (3–6)	<0.00001	4.74 ± 0.89 (3–6)	0.000096

**Figure A1:**
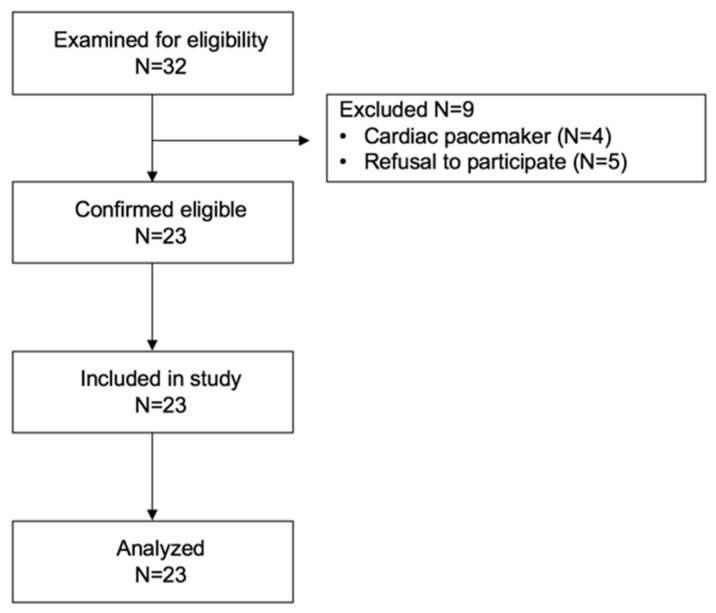
Patient inclusion flowchart.

**Figure A2:**
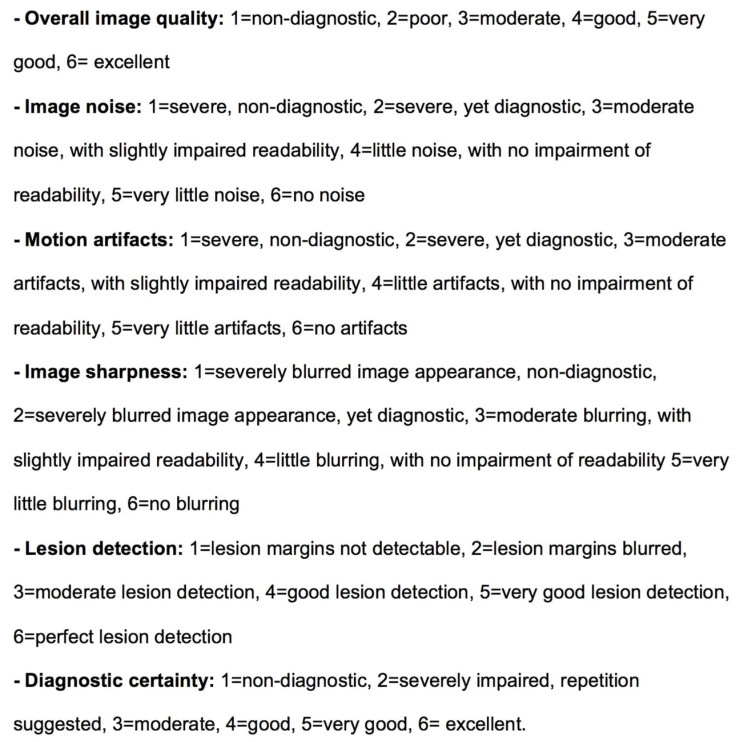
Detailed Likert-scale parameters.

## Data Availability

Data are available on request.

## Abbreviations

ADC: Apparent diffusion coefficient, aCNR: Apparent contrast-to-noise ratio, aSNR: Apparent signal-to-noise ratio, CS: Compressed sensing, CNN: Convolutional Neural Network, C-SENSE: Compressed SENSE, DL: Deep learning, DnCNN: Denoising convolutional neuronal network, DW: Diffusion-weighted, DWI: Diffusion-weighted imaging, FOV: Field of view, GPU: Graphics processing unit, HBB: Hyoscine butylbromide, ISTA: Iterative Shrinkage–Thresholding Algorithm, PCa: Prostate cancer, PI: Parallel imaging, PI-RADS: Prostate imaging-reporting and data system, mpMRI: Multiparametric magnetic resonance imaging, MRI: Magnetic resonance imaging, NSA: Number of signal averages, PSA: Prostate-specific antigen, ROI: Region of interest, SD: Standard deviation, SI: Signal intensity, SNR: Signal-to-noise ratio, ssEPI: Single-shot echo planar imaging, T2w: T2-weighted, TSE: Turbo-spin echo.
